# Community-led intensive trapping reduces abundance of key plague reservoir and flea vector

**DOI:** 10.1186/s41182-025-00746-0

**Published:** 2025-05-09

**Authors:** Marcela P. A. Espinaze, Soanandrasana Rahelinirina, Todisoa Radovimiandrinifarany, Fehivola Mandanirina Andriamiarimanana, Alain Berthin Andrianarisoa, Voahangy Soarimalala, Kathryn Scobie, Mireille Harimalala, Minoarisoa Rajerison, Steven R. Belmain, Sandra Telfer

**Affiliations:** 1 School of Biological Sciences, University of Aberdeen, Zoology Building, Tillydrone Avenue, Aberdeen, AB24 2TZ Scotland, UK; 2 Plague Unit, Institut Pasteur de Madagascar, Antananarivo, Madagascar; 3 Ecole Doctorale Sciences de la Vie et de l’Environnement, University of Antananarivo, Antananarivo, Madagascar; 4 Association Vahatra, Antananarivo, Madagascar; 5 Institut des Sciences et Technique de l’Environnement, University of Fianarantsoa, Fianarantsoa, Madagascar; 6 Unité d’Entomologie Médicale, Institut Pasteur de Madagascar, Antananarivo, Madagascar; 7 Natural Resources Institute, University of Greenwich, Kent, UK

**Keywords:** Plague, Siphonaptera, Rodent-borne diseases, Zoonosis, Rural population, Rodent control, Madagascar

## Abstract

**Background:**

Zoonotic pathogens transmitted by rodents are highly prevalent in low-middle income countries and effective control measures that are easily implemented are urgently needed. Whilst rodent control seems sensible as a mitigation strategy, there is a risk that disease prevalence in reservoir populations can increase following control due to impacts on movement and demographics. Additionally, removing rodents from the population does not necessarily lead to reductions in abundance as populations can compensate for removal through increased breeding and immigration. In a previous study of intermittent control within houses, we showed that reduction in rodent abundance was only very short-term. Working in rural settings within the plague-endemic area of Madagascar, this study explores whether community-led daily intensive rodent trapping within houses can effectively reduce long-term rodent and flea abundance.

**Main text:**

A rodent management experiment was carried out in six rural villages of Madagascar during 2022–2023. Three villages were selected as intervention villages, where intensive daily rodent trapping inside houses was conducted. Surveillance of rodent and flea abundance using traps and tiles took place at 4-month intervals. We show that community-led intensive rodent trapping in rural Malagasy households effectively reduced abundance of the main rodent reservoir (*Rattus rattus*) and indoor flea vector (*Xenopsylla cheopis*) of plague. Importantly, indoor abundance of the outside flea vector (*Synopsyllus fonquerniei*) did not increase.

**Conclusions:**

Community-based intensive rodent trapping inside houses is an effective methodology in controlling key reservoirs and vectors of plague, which can be implemented by the communities themselves. Co-ordinated and sustained rodent control should be considered as an important plague mitigation strategy.

**Supplementary Information:**

The online version contains supplementary material available at 10.1186/s41182-025-00746-0.

## Background

Rodents are important hosts of zoonotic pathogens, including plague which is caused by the bacteria *Yersinia pestis* [1]. In Madagascar, where 81% of global plague cases occurred between 2013 and 2018 [2], most cases occur in the central highlands [3]. The main epidemiological cycle involves black rats (*Rattus rattus*) and two flea species, *Xenopsylla cheopis*, which parasitizes rats inside houses, and *Synopsyllus fonquerniei*, which is prevalent on outside rats and the distribution of which closely matches that of human plague cases, highlighting its importance [4]. Plague mitigation strategies largely focus on preventing transmission to humans and, therefore, the human–flea interface, whilst the risk posed by infected fleas leaving dead rodents has led to mixed advice on the value of rodent control [5]. Nevertheless, since reducing rodent abundance can lead to vector population reductions, effective rodent management could be an important strategy in tackling plague risk [3].

On the face of it, localized control seems a sensible strategy for reducing rodent abundance in and around human habitation; however, rats are well known for being neophobic (avoiding new things in their environment), and capable of compensatory reproduction and immigration which can counteract the effects of control. Indeed, in a previous study of intermittent control within houses we showed that reduction in abundance was only very short-term [3]. Studies elsewhere have found similar results [6]. Furthermore, although some studies have shown that intensive community-led trapping can be effective at reducing rodent populations in rural African households, most previous research has focussed on food security benefits [7, 8], whereas others have shown that spatially limited control can potentially exacerbate disease transmission within reservoirs due to increased infected host movement towards areas populated by susceptible hosts [9, 10] and possibly by changes in population demographics and disease susceptibility [11]. Therefore, the complex and context-specific epidemiology of rodent-borne diseases necessitates careful evaluation of control measures targeting rodents and/or vectors [5].

In rural Madagascar, black rats are prevalent both inside houses and outside, in peri-domestic areas and in agricultural fields. Studies have shown that there is migration of black rats between these habitats. We are therefore faced with the challenge that removal efforts inside houses may be compensated for through immigration. Furthermore, there are major concerns that increased immigration may increase exposure to the outside flea *S. fonquerniei*, potentially increasing plague exposure risk to people. We addressed these knowledge gaps by assessing the impact of intensive trapping inside households on key indices of plague risk: rodent abundance, rodent flea infestation (i.e. proportion of rodents that carry a flea), and flea abundance.

## Main text

We conducted a rodent management experiment in six villages in Analavory Commune-Miarinarivo District-Itasy Region, Madagascar, during 2022–2023 (Additional file 1: Figure S1, Table 1). In three (intervention) villages, communities themselves conducted intensive daily rodent trapping inside all houses. Two traps were distributed per household, with snap-traps (Romax Snap-R, 14 L × 7.5 W × 6.5 H cm) used during the non-plague season (May–August 2022) and live-capture wire mesh traps (BTS Company 30 L × 10 W × 10 H cm) used during the plague season (September 2022–April 2023). Community agents recorded the number of rodents captured per day. Live-caught rodents were euthanized by cervical dislocation. In three (control) villages, householders could continue any rodent management activities they usually used. Monitoring of rodent and flea abundance was conducted at 4-month intervals (March 2022–March 2023) within 16 houses per village in four sampling areas, using tracking tiles and trapping inside houses. Tracking tiles recorded activity (rodent footprints, scratches and tail swipes) using ceramic tiles (20 × 20 cm) painted with a mixture of 32% blue chalk powder, 64% white spirit, and 4% motor oil [3]. Three tracking tiles were distributed in each house and checked daily, with tiles set for two nights in July and November 2022 and one night in March 2023. After tiles were removed, one live-capture wire mesh trap (30 L × 10 W × 10 H cm) and one Sherman trap (H.B. Sherman Traps Inc., 23 L × 7.5 W × 9 H cm) were set in each house for four nights (reduced to two nights in March 2023) and checked daily. Captured animals were euthanized by cervical dislocation and identified to species. Animals were brushed to remove fleas which were stored in 95% alcohol and later identification was performed using a binocular microscope (Leica, Wetzlar, Germany) and available morphological keys [12]. Blood samples were collected through cardiac puncture. Whole blood was centrifuged to separate the serum and kept at 4 °C in the field and − 20 °C on return to laboratory. Detection of anti-F1 IgG antibodies was performed using an enzyme-linked immunosorbent assay (ELISA), with samples tested in duplicate and a mean optical density of 0.15 used as a threshold for IgG detection [13]. In each plate a negative and a positive rodent sample were included as controls.

**Table 1:** Intervention status, number of households and coordinates of the villages selected for this study

Village name	Village code	Intervention	No. households	Latitude	Longitude
Ambohitrakoho	AMB	Yes	70	− 18.957351	46.731569
Ambohitsaratelo	ATL	Yes	50	− 18.926806	46.652556
Mahiatrondro ambony	MHH	Yes	232	− 19.021285	46.714435
Bengitsy	BEN	No	131	− 18.934844	46.718157
Soanafindra	SOA	No	97	− 18.892869	46.661715
Amparihy	APR	No	88	− 19.025972	46.68675

Generalized Linear Mixed Models were used to test for differences between control and intervention villages in: (1) rodent (*R. rattus* and *Mus musculus*) relative abundance from capture and tracking tile data; (2) probability of being infested by a flea (for each flea species separately); and (3) relative abundance of each flea species. Flea analyses focussed on data from *R. rattus* as *M. musculus* carried few fleas. We checked for differences between intervention and control villages prior to the experiment, using data from March 2022 for rodents and *X. cheopis* and, due to seasonality of *S. fonquerniei* [4], data from pilot sampling conducted in August–October 2021 (peak abundance period for this flea). Intervention analyses examined the effectiveness of treatment using monitoring data from July 2022–March 2023. Rodent and flea abundance can vary seasonally, whilst the effectiveness of management may accumulate over time or differ seasonally due to changes in rodent reproduction or movement [1, 14]. Therefore, intervention period analyses evaluated month and treatment, either individually, additively or with an interaction. For count analyses, the response was captures per household with an offset of sampling effort (calculated as the number of traps containing rodents or not sprung plus half the number of traps which were sprung or had bait removed but which had not caught a rodent) [15]. We used Akaike’s information criterion (AIC) to compare Poisson, negative binomial, zero-inflated Poisson and zero-inflated negative binomial models, selecting the distribution with the lowest AIC. For analyses of tracking tile and flea infestation data, presence or absence per tile or per rat, respectively, was modelled with a binomial distribution. To capture spatial variation not related to treatment, models included random effects of house (for intervention analyses), nested in sampling area, nested in village. We present treatment effects from the model with the lowest AIC that included treatment. Models were run using the glmmTMB package in R software (version 4.2.0; R Foundation for Statistical Computing, Vienna, Austria, https://cran.r-project.org/).

**Fig. 1:**
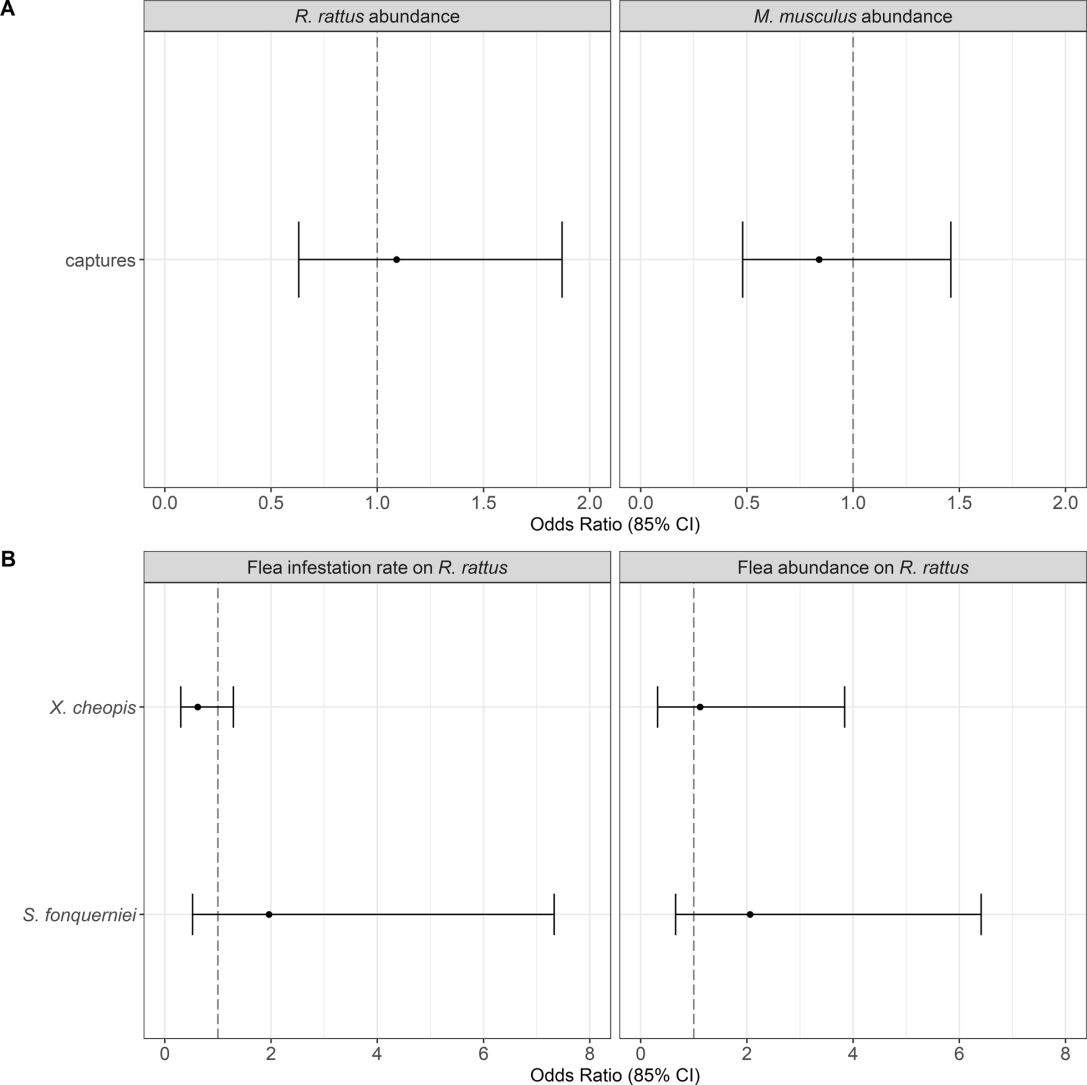
**A** Effect of treatment on the relative abundance of *R. rattus* and *M. musculus* inside houses during the pre-intervention period (March 2022). Data were collected using trapping (captures). There was no data collected from tracking tiles during the pre-treatment period. Models for *R. rattus* and *M. musculus* used a negative binomial and Poisson distribution, respectively. **B** Effect of treatment on the probability of being infested by and relative abundance of *X. cheopis* and *S. fonquerniei* on *R. rattus* inside houses during the pre-intervention period (March 2022 for *X. cheopis* and August or October 2021 for *S. fonquerniei*). Models for the probability of being infested by a flea species used a binomial distribution, and models for relative abundance used a negative binomial distribution for both flea species. The forest plots illustrate the odd ratios (circles) and 85% confidence intervals (CI confidence interval; whiskers) for the effect of treatment. Confidence intervals overlap 1 (vertical dotted line) indicating no difference between intervention and non-intervention villages during the pre-intervention period. For all analyses, the model with the lowest AIC was the intercept only model

**Fig. 2:**
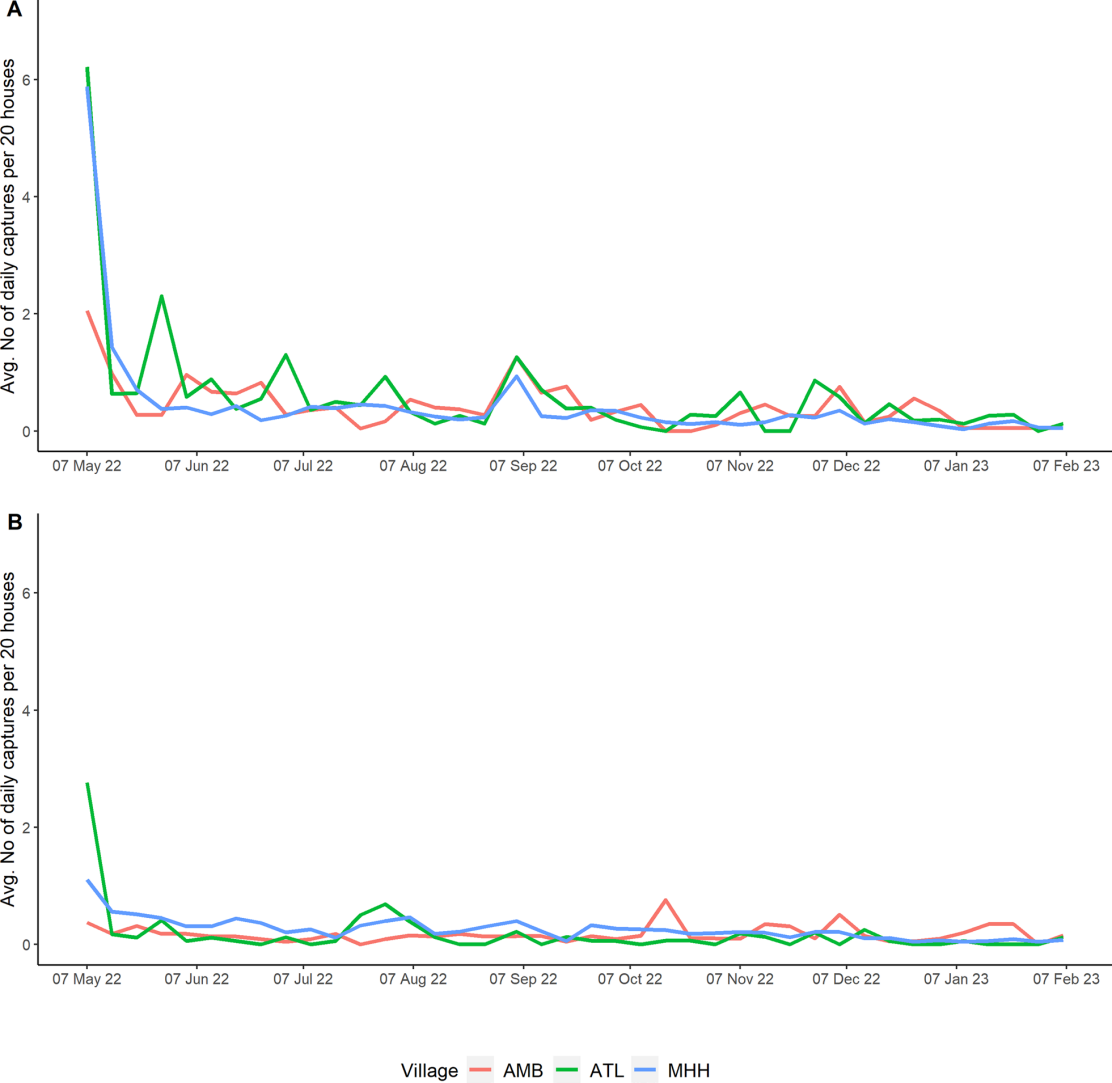
Results from the daily trapping in each of the three intervention villages between May 2022 (i.e. the start of daily trapping) and February 2023. Plots show weekly averages for the number of (**A**) rats and (**B**) mice caught by daily trapping, standardised to reflect numbers per 20 houses. Live-capture traps were used from September 2022 to February 2023 (i.e. during the plague season)

**Fig. 3:**
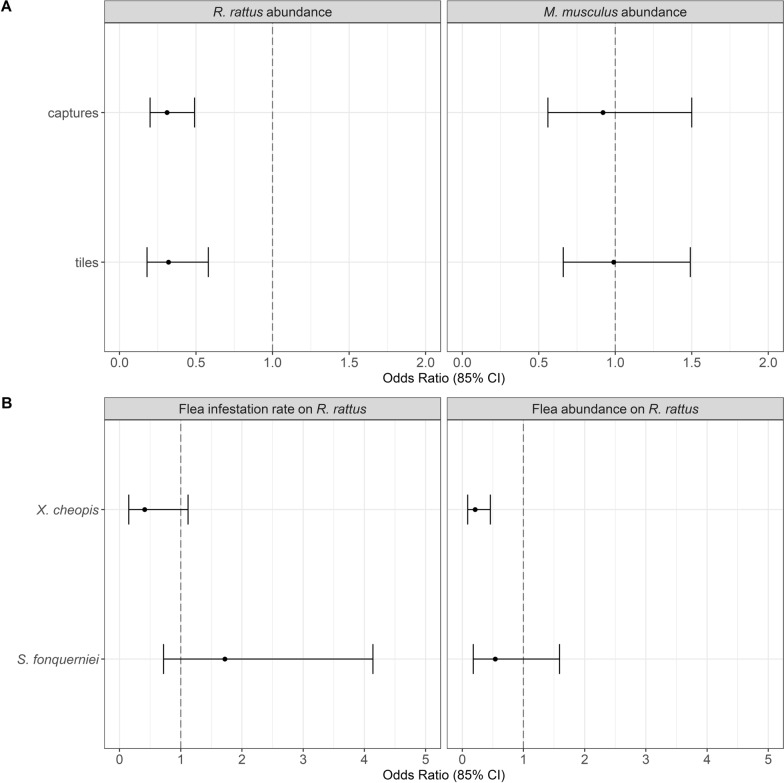
**A** Effect of treatment on the relative abundance of *R. rattus* and *M. musculus* inside houses during the intervention period (July 2022-March 2023). Data were collected using trapping (captures) and tracking tiles. Effects based on the model with the lowest AIC that included treatment. Models for capture data of *R. rattus* and *M. musculus* used a Poisson and negative binomial distribution, respectively. Models for tracking tile data used a binomial distribution for both rodent species. **B** Effect of treatment on the probability of being infested by and relative abundance of *X. cheopis* and *S. fonquerniei* on *R. rattus* inside houses during the intervention period. Effects based on the model with the lowest AIC that included treatment. Models for the probability of being infested by a flea species used a binomial distribution for both flea species. Models for relative abundance of *X. cheopis* and *S. fonquerniei* used a negative binomial and a Poisson distribution, respectively. Forest plot illustrates the odd ratios (circles) and 85% confidence intervals (CI confidence interval; whiskers). Effects with confidence intervals overlapping 1 (vertical dotted line) indicate no significant treatment effect (treatment was a non-informative parameter), whilst those with confidence intervals not overlapping 1 indicate a significant effect of treatment

Preliminary analysis confirmed that, prior to our treatment, intervention and control villages did not differ in rodent or flea abundances (Table 2, Fig. 1). Between end April 2022 and end February 2023, 2013 *R. rattus* and 1297 M*. musculus* were removed by daily trapping from the three intervention villages, with daily captures declining rapidly and then remaining low (Fig. 2). Evidence from monitoring indicated that treatment effectively reduced *R. rattus* abundance inside houses, but not *M. musculus* (Fig. 3A, Table 2). There was evidence that *R. rattus* were less likely to be infested by *X. cheopis* in intervention villages, and this tendency combined with the large decline in the numbers of *R. rattus* led to a considerable decline in the relative abundance of *X. cheopis* in intervention villages (Fig. 3B). Importantly, we found no evidence that inside house rats in intervention villages were more likely to be infested with *S. fonquerniei*, the flea typically found on outside rats [4], nor that its relative abundance was greater inside houses in intervention villages (Fig. 3B). Moreover, the total flea index, a commonly used measure of risk in plague studies [3], was reduced by 46% in intervention villages compared to control villages (Table 2). Our analyses also highlighted seasonal variation in *R. rattus* and *M. musculus* abundance. We found little evidence of interactions between treatment and month except for tracking tile data for *R. rattus*, where there was evidence that the inside house rat population in intervention villages partially recovered in March 2023 (Tables 3 and 4). The 133 *R. rattus* with serum samples were all seronegative.

**Table 2:** Rodent captures, on-rodent flea abundance, rodent flea infestation and flea index at intervention and control villages before and during intervention

Parameters	Pre-intervention		Intervention		
	Intervention villages	Control villages	Intervention villages	Control villages	Reduction rate^d^
*Rattus rattus* captures^a^	11.33 ± 1.33 (34)	10.33 ± 3.48 (31)	3.78 ± 0.8 (34)	11.56 ± 2.87 (104)	− 67
*Mus musculus* captures^a^	4.67 ± 0.88 (14)	5.67 ± 1.76 (17)	8.78 ± 2.68 (79)	9.67 ± 2.26 (87)	− 9
*Infestation of Xenopsylla cheopis* on *rats*^b^	0.54 ± 0.06	0.67 ± 0.04	0.12 ± 0.05	0.32 ± 0.05	− 63
*Infestation of Synopsyllus fonquerniei* on rats^b^	0.19 ± 0.12	0.13 ± 0.07	0.16 ± 0.05	0.12 ± 0.04	28
*Xenopsylla cheopis* abundance on rats^b^	53.67 ± 30.72 (161)	47.67 ± 22.42 (143)	1.44 ± 0.88 (13)	7.0 ± 1.38 (63)	− 79
*Synopsyllus fonquerniei* abundance on rats^b^	4.67 ± 3.28 (14)	2.67 ± 1.45 (8)	1.0 ± 0.53 (9)	1.44 ± 0.44 (13)	− 31
*Total flea index on rats*^*c*^	4.71 ± 1.92	5.05 ± 1.89	0.48 ± 0.20	0.89 ± 0.20	− 46

^a^Rodent (*Rattus rattus* and *Mus musculus*) captures during pre-intervention period took place in March 2022 and during intervention period took place in July 2022, November 2022 and March 2023. Data are presented as the average captures across all sites and sampling months ± standard error, and the total number in brackets

^b^Collection of *Xenopsylla cheopis* fleas on rodents during pre-intervention took place in March 2022, and collection of *Synopsyllus fonquerniei* fleas during pre-intervention took place in August or October 2021. Intervention period sampling took place in July 2022, November 2022 and March 2023. Only data for rats are presented as few fleas were collected from mice (*n* = 20 in total). Rodent flea infestation is the proportion of rodents that carry fleas (probability of being infested with fleas), with data presented as the average across all sites and sampling months ± standard error. Flea abundance data are presented as the average abundance across all sites and sampling months ± standard error, and the total number in brackets. High abundance of *X. cheopis* in the pre-intervention period in one intervention village and one control village contributes to high average values and standard errors

^c^Flea index = total number of fleas/number of rats. Data are presented as the average across all sites and sampling months ± standard error

^d^Reduction rate = percentage change in captures, flea infestation, flea abundance and flea index at intervention sites compared to control sites during intervention period using the average values. It was calculated as ((intervention—control)/control)*100

**Table 3:** Final competitive model(s) for rodent relative abundance inside houses during the intervention period (July 2022-March 2023)

Level	Variable	*Rattus rattus* relative abundance^a^				*Mus musculus* relative abundance^a^		
		Captures (model 1^b^)	Captures (model 2^b^)	Tracking tiles (model 1^c^)	Tracking tiles (model 2^c^)	Captures (model 1^d^)	Captures (model 2^d^)	Tracking tiles (model 1^c^)
Intervention period^e^	Intercept	0.08 (0.06–0.1)	0.09 (0.06–0.12)	1.43 (0.85–2.4)	2.08 (1.09–4.0)	0.17 (0.13–0.22)	0.19 (0.14–0.26)	1.64 (1.16–2.31)
	Treatment (yes)	**0.31 (0.20–0.49)**	**0.31 (0.20–0.49)**	**0.32 (0.18–0.58)**	**0.16 (0.06–0.4)**			
	Month (Nov 2022)		**0.7 (0.53–0.92)**	**0.38 (0.21–0.68)**	**0.27 (0.12–0.62)**		0.94 (0.70–1.26)	**2.28 (1.35–3.85)**
	Month (March 2023)		0.88 (0.63–1.24)	**0.55 (0.32–0.93)**	**0.27 (0.12–0.6)**		**0.62 (0.41–0.93)**	**0.61 (0.39–0.96)**
	Treatment (yes)* Month (Nov 2022)				1.72 (0.54–5.47)			
	Treatment (yes)* Month (March 2023)				**3.99 (1.34–11.86)**			
Random effects^f^	House: Sampling area: Village	0.92 (0.96)	0.91 (0.96)	1.36 (1.17)	1.5 (1.22)	< 0.001 (< 0.001)	< 0.001 (< 0.001)	1.57 (0.4)
	Sampling area: Village	0.02 (0.15)	0.03 (0.17)	0.03 (0.17)	0.05 (0.22)	0.03 (0.2)	0.03 (0.2)	< 0.001 (< 0.001)
	Village	< 0.001 (< 0.001)	< 0.001 (0.006)	< 0.001 (< 0.001)	< 0.001 (< 0.001)	0.11 (0.34)	0.12 (0.34)	< 0.001 (< 0.001)

^a^We present odd ratios and 85% confidence intervals from the final competitive model(s), and variance and standard deviation of random effects. Final competitive models for each analysis included the model with the lowest AIC and any models with an AIC within 2, excluding models which only differed by the inclusion of uninformative parameters based on 85% confidence intervals (Leroux SJ. On the prevalence of uninformative parameters in statistical models applying model selection in applied ecology. PLoS One. 2019;14:p.e0206711. https://doi.org/10.1371/journal.pone.0206711). Informative parameters are highlighted in bold

^b^Models used a Poisson distribution

^c^Models used a binomial distribution

^d^Models used a negative binomial distribution

^e^In order, the intervention period row includes odds ratios and 85% confidence intervals (in brackets) for each variable

^f^ In order, the random effects row displays the variance and standard deviation (in brackets) for each variable

**Table 4:** Final competitive model(s) for the probability of being infested by a flea species and relative abundance of rodent fleas inside houses during the intervention period (July 2022-March 2023)

Level	Variable	*X. cheopis* infestation on* R. rattus*^ab^	*S. fonquerniei* infestation on* R. rattus*^ab^	*X. cheopis* abundance on *R. rattus*^ac^	*S. fonquerniei* abundance on* R. rattus*^ad^
Intervention period^e^	Intercept	0.28 (0.17–0.46)	0.15 (0.09–0.23)	0.07 (0.04–0.12)	0.00 (0.00–0.01)
	Treatment (yes)			**0.21 (0.09–0.46)**	
	Month (Nov 2022)				**0.22 (0.09–0.54)**
	Month (March 2023)				0.89 (0.42–1.89)
	Treatment (yes) * Month (Nov 2022)				
	Treatment (yes) * Month (March 2023)				
Random effects^f^	House: Sampling area: Village	< 0.001 (< 0.001)	< 0.001 (< 0.001)	< 0.001 (< 0.001)	4.84 (2.2)
	Sampling area: Village	0.52 (0.72)	< 0.001 (< 0.001)	< 0.001 (< 0.001)	< 0.001 (0.01)
	Village	0.15 (0.39)	0.14 (0.37)	< 0.001 (< 0.001)	< 0.001 (0.007)

^a^We present odd ratios and 85% confidence intervals from the final competitive model(s), and variance and standard deviation of random effects. Final competitive models for each analysis included the model with the lowest AIC and any models with an AIC within 2, excluding models which only differed by the inclusion of uninformative parameters based on 85% confidence intervals (Leroux SJ. On the prevalence of uninformative parameters in statistical models applying model selection in applied ecology. PLoS One. 2019;14:p.e0206711. https://doi.org/10.1371/journal.pone.0206711). Informative parameters are highlighted in bold

^b^Models used a binomial distribution

^c^Models used a negative binomial distribution

^d^Models used a Poisson distribution

^e^In order, the intervention period row includes odds ratios and 85% confidence intervals (in brackets) for each variable

^f^In order, the random effects row displays the variance and standard deviation (in brackets) for each variable

## Conclusions

Our results show that intensive trapping inside houses can reduce rodent populations and maintain low numbers, and significantly reduce the abundance of a key flea vector of plague (*X. cheopis*). These results highlight that communities working together can impact the risk from rodent-borne diseases, despite the high reproduction potential of rodents. Furthermore, in the context of plague epidemiology in Madagascar, we found no evidence that removing rats from inside houses had an effect on the abundance of *S. fonquerniei* inside houses. Thus, there is no evidence of counterproductive effects of intensive trapping on vector movement and disease transmission, such as was found for Lassa virus infection following intensive rodent trapping in Guinea, West Africa [11]. This is of major concern for health authorities in Madagascar, where the human plague season takes place from September to April. The start of this period appears to coincide with high abundances of *S. fonquerniei* on *R. rattus* outside houses, with previous studies indicating high abundances from September to January, peaking in October [16]. There is therefore apprehension about movement of *R. rattus* carrying *S. fonquerniei* into houses from surrounding areas [3]. Our findings therefore have important implications for plague mitigation strategies in Madagascar.

In terms of *R. rattus* abundance, the effects of community-led intensive trapping were rapid rather than cumulative. However, the slight recovery of house rat populations in March may reflect immigration following the peak reproduction period for rats outside [6]. This further emphasizes the need for sustained control in order to overcome the compensatory responses of rodent populations. We therefore recognize that the duration of interventions must be extended to keep rodent abundance low and prevent their increase as has occurred in short-term interventions [3, 6]. We believe the lack of an effect on *M. musculus* populations is related to trap type, with the trigger mechanism being less sensitive to smaller-sized animals; additional studies are assessing whether smaller snap-traps and live-capture traps with finer wire mesh may be more effective at removing these individuals. Finally, in follow-up surveys, participating communities expressed their commitment to continuing community-led intensive rodent trapping, whilst local authorities indicated a desire for the strategy to be expanded to all villages within the commune. Maintaining community engagement and motivation will be crucial in determining the long-term sustainability of this approach, whilst it will also be important to evaluate how social and economic aspects influence its applicability to other contexts.

## Supplementary Information

**Additional file 1: Figure S1.** Map of the study area in Madagascar, showing the location of treatment and non-treatment villages, as well as the approximate area of the main endemic plague focus in the Central highlands of Madagascar.

## Data Availability

The datasets used and/or analysed during the current study are available from the corresponding author on reasonable request.

## Abbreviations

ELISA: Enzyme-linked immunosorbent assay
AIC: Akaike’s information criterion
CI: Confidence interval
OD: Optical density
