# Supplementary figures and images for: Community-led intensive trapping reduces abundance of key plague reservoir and flea vector
Source: Trop Med Health. 2025 May 9;53:67. doi: 10.1186/s41182-025-00746-0 (PMC12063217; doi:10.1186/s41182-025-00746-0)

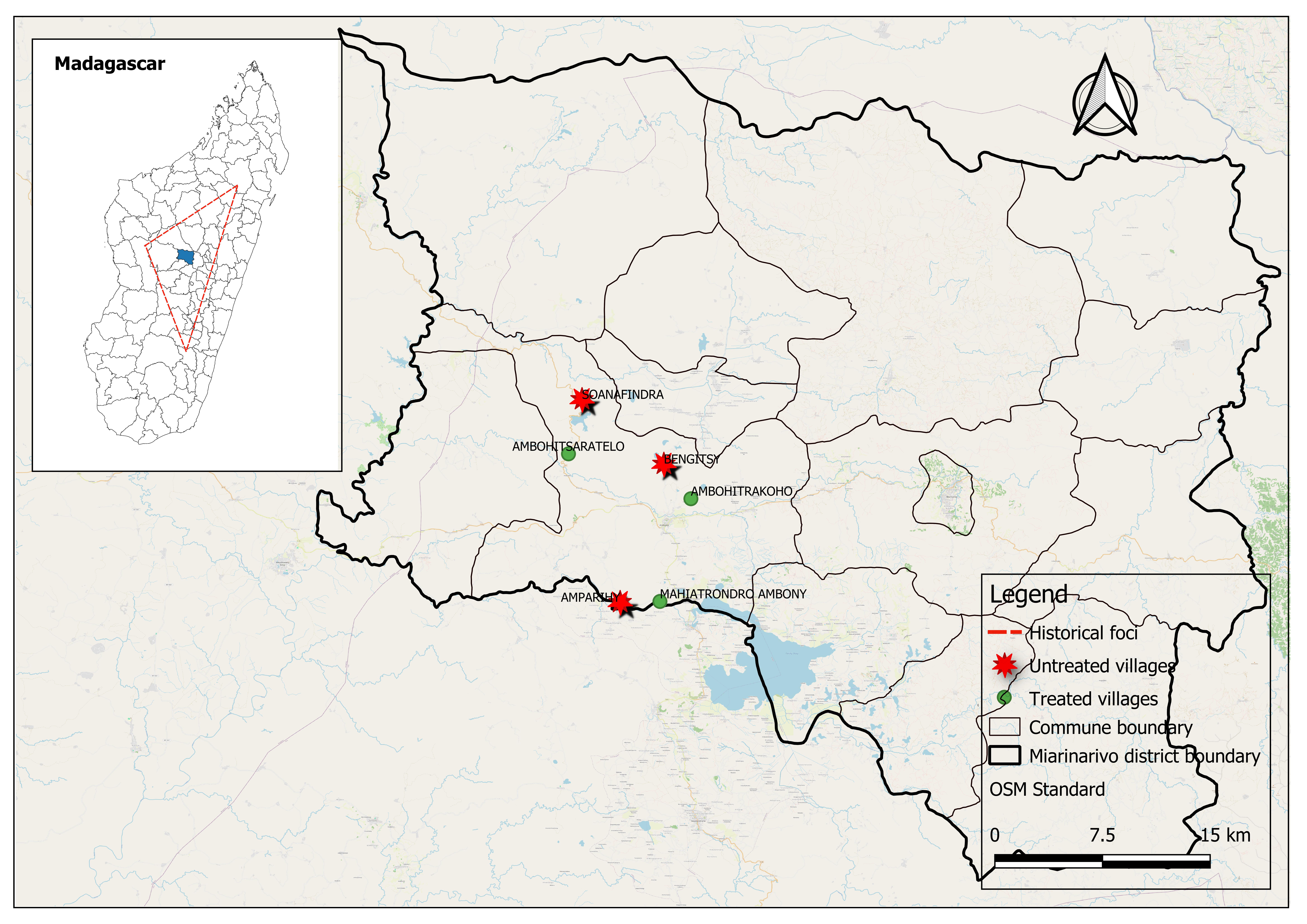

Supplement: Supplementary file 1 — Additional file 1: Figure S1. Map of the study area in Madagascar, showing the location of treatment and non-treatment villages, as well as the approximate area of the main endemic plague focus in the Central highlands of Madagascar. [file 41182_2025_746_MOESM1_ESM.jpeg]
